# Papillary renal neoplasm with reverse polarity may be a novel renal cell tumor entity with low malignant potential

**DOI:** 10.1186/s13000-022-01235-2

**Published:** 2022-08-25

**Authors:** Tong Yang, Enhao Kang, Longxiao Zhang, Jie Zhuang, Yujun Li, Yanxia Jiang, Han Wang, Wenjuan Yu, Wei Zhang

**Affiliations:** 1 Department of Pathology, No.971 Hospital of People’s Liberation Army Navy, No.22, Minjiang Road, Qingdao, 266071 Shandong China; 2 Department of Pathology, The Affiliated Hospital of Qingdao University, No.16, Jiangsu Road, Qingdao, 266003 Shandong China

**Keywords:** Neoplasm, renal cell, Papillary, Oncocyte, Reverse polarity, *KRAS*

## Abstract

**Aims:**

This study retrospectively investigated the morphological, immunohistochemical and molecular genetic features of papillary renal neoplasm with reverse polarity (PRNRP), a recently described renal tumor.

**Methods and results:**

Eleven cases of PRNRP were collected, and 16 cases of type I and 9 cases of type II papillary renal cell carcinoma were included as a control series. Pathological features were evaluated based on HE staining and immunohistochemistry. *KRAS* exon 2 and *BRAF* V600E mutations were detected by Real-time PCR and Sanger sequencing. Fluorescence in situ hybridization was conducted for identification of chromosomal abnormalities.

Hemosiderin deposition was found in a small amount of tumor cells in 6 cases. Multifocal or patchy necrosis (5/11), small focal invasion of the pseudocapsules or renal parenchyma (6/11), and breakthrough of renal capsule with nerve invasion (1/11) were revealed, inconsistent with the previous view that the tumor lacks necrosis and intercellular hemosiderin. Immunohistochemical staining (diffusely positive for CK7 and GATA3, negative for CD117 and vimentin, and negative to weakly positive for P504S) and high frequency of *KRAS* mutations in exon 2 (9/10) supported the identification and inclusion of our cases. Chromosome 7 trisomy (1/7), chromosome 17 trisomy (0/7) and chromosome Y deletion (0/5 male patients) were seldom detected in this tumor.

All patients were alive without metastasis or recurrence at the end of the follow-up.

**Conclusion:**

Our findings may highlight the possibility of a low malignant potential of this emerging entity. We suggest that the tumor be classified as a novel renal cell tumor subtype independent of papillary renal cell carcinoma.

**Supplementary Information:**

The online version contains supplementary material available at 10.1186/s13000-022-01235-2.

## Introduction

Renal cell carcinoma (RCC) is a comprehensive concept, which includes a variety of tumor entities with independent driving factors, histological patterns and clinical prognosis. Papillary RCC (PRCC) accounts for 15% to 20% of RCC [1], with a second highest incidence rate in all renal cell carcinoma, next only to clear cell RCC (CCRCC). In 1997, Delahunt and Able [2] classified PRCC into type I and type II according to their morphological characteristics. Type I PRCC typically has relatively small cells with scant basophilic cytoplasm and uniform small oval nucleus; while in type II PRCC, the papillary structures are usually covered by larger cells with abundant eosinophilic cytoplasm and large round nuclei with obvious nucleoli. This dichotomy is of important clinical value, since compared with type I PRCC patients, patients with type II PRCC usually have advanced tumor stage and worse prognosis [3]. However, with the deepening of research, increasing evidence suggests that type II PRCC may contain a spectrum of heterogenous subtypes. Due to the development of immunohistochemistry and molecular techniques, the classification of RCC has been further enriched and refined. Several renal tumors with papillary morphology have been identified and classified into independent subtypes, such as hereditary leiomyomatosis and renal cell carcinoma-associated RCC (HLRCC-RCC) [4], succinate dehydrogenase-deficient RCC [5], MiT family translocation RCC [6], etc.

Papillary renal neoplasm with reverse polarity (PRNRP) [7], also called oncocytic papillary renal neoplasm with inverted nuclei (OPRNIN) [8], is a newly recognized renal tumor in recent years. Morphologically, it is characterized by papillary or tubular structure covered by a single layer of cuboidal to columnar eosinophilic cells with nuclei located at the apical surface far away from the basement membrane. Because of the limited reports on this tumor, its clinicopathological characteristics, immunophenotype, biomolecular features and the relationship between PRNRP and classical type I and type II PRCC still remain to be further explored. Previously, PRNRP was regarded as a low-grade renal tumor with a favorable prognosis, and intracellular hemosiderin and necrosis were described to be absent [7–9]. However, we retrospectively analyzed 11 cases of PRNRP and found necrosis and hemosiderin deposition, as well as histological features of local invasion of peripheral nerves and renal parenchyma in this tumor entity. Given that the tumor exhibits a papillary morphology but only a minimal proportion of PRNRP was confirmed to present chromosome 7 trisomy, chromosome 17 trisomy or chromosome Y deletion, we suggest that it be classified as an independent subtype of renal cell neoplasm, distinguishable from PRCC.

## Materials and methods

### Data collection

A total of 11 cases of PRNRP diagnosed by pathological examination were collected from No.971 Hospital of People's Liberation Army Navy (4 cases, including 1 consultation case) and the Affiliated Hospital of Medical College, Qingdao University (7 cases) between 2010 and 2018. All the hematoxylin and eosin (HE) and immunohistochemical staining results were independently assessed by at least 2 senior pathologists. Clinical data were obtained through review of electronic medical records, and the gross morphology information was from pathological dissection records. Nuclear grading was performed according to the WHO/ISUP system [10]. Grade 1 and 2 were classified as low grade and Grade 3 and 4 were high grade. TNM stage was determined based on the eighth edition of the American Joint Committee on Cancer (AJCC) staging manual [11] and grouped as early-stage (stage I and II) or advanced-stage (stage III and IV). Survival status of patients were followed up by telephone. In addition, 16 cases of type I and 9 cases of type II PRCC were included as a control series. The study was approved by the medical ethics review committee of the corresponding institutions.

### HE and immunohistochemical staining

All the specimens were fixed with 3.7% neutral formaldehyde, dehydrated, paraffin embedded, cut into 5 μm sections and HE stained. Immunohistochemical staining was performed on a VENTANA BenchMark XT automated staining system (Ventana Medical Systems, Inc., Tucson, AZ, USA). Primary antibodies against vimentin, epithelial membrane antigen (EMA), cytokeratin 7 (CK7), CD10, CD117, carbonic anhydrase-IX (CA9), a-methylacyl-coenzyme A racemase (AMACR/P504S), GATA3, renal cell carcinoma marker (RCC), paired box protein 8 (pax-8), transcription factor E3 (TFE3), anaplastic lymphoma kinase (ALK) and Ki-67 were purchased from MXB Biotechnologies, Fuzhou, China, and positive and negative controls were also performed for each antibody.

### Real-time fluorescent quantitative polymerase chain reaction (real-time PCR) and Sanger sequencing

Mutations in *KRAS* and *BRAF* were detected by means of real-time PCR and Sanger sequencing. A detailed description of mutation detection is presented in the Supplementary materials and methods.

### Fluorescence in situ hybridization (FISH)

FISH analysis was performed to identify chromosome 7 trisomy, chromosome 17 trisomy and chromosome Y deletion in PRNRP cases. For detailed information, see Supplementary materials and methods.

### Statistical analysis

SPSS software (version 19.0; SPSS, Chicago, IL, USA) was used for statistical analyses. One-way ANOVA test followed by Tukey’s multiple comparisons test were conducted for multiple comparisons of continuous data. Proportional differences were evaluated by Chi-square test or Fisher’s exact probability test, if appropriate. All statistical tests were two-tailed, and a *P-value* less than 0.05 was required for statistical significance.

## Results

### Clinical data

Among the 11 PRNRP cases, 7 were male and 4 were female, ranging in age from 26 to 70 years (mean age 58 years). Nine cases (82%) were found by physical examination, and 2 originally presented with continuous lumbago and left lower abdominal pain, respectively. Seven tumors were located in the left kidney and 3 in the right kidney. A 60-year-old male patient (Case 11) presented with bilateral kidney tumors. On CT scans, most of the tumors were round or nodular hypodense shadows in renal cortex near the kidney capsule, heterogeneous in density. Five patients had tumors protruding from the surface of the kidney, including 1 patient with a multifocal tumor, 3 patients with multiple renal cysts, and 1 patient (Case 3) with nodular medium-density shadow in the ipsilateral adrenal gland. TNM stage was stage 1 for all PRNRP patients (9 were pT1aN0M0, 2 was pT1bN0M0). Partial nephrectomy was performed on 9 patients (81.8%), and the remaining 2 patients (18.2%) underwent radical nephrectomy (Case 3 combined with partial right adrenalectomy). No significant difference was found in age, sex ratio or TNM stage between patients with PRNRP, type I or type II PRCC (*P* > 0.05).

### Histomorphological investigations

**Fig. 1:**
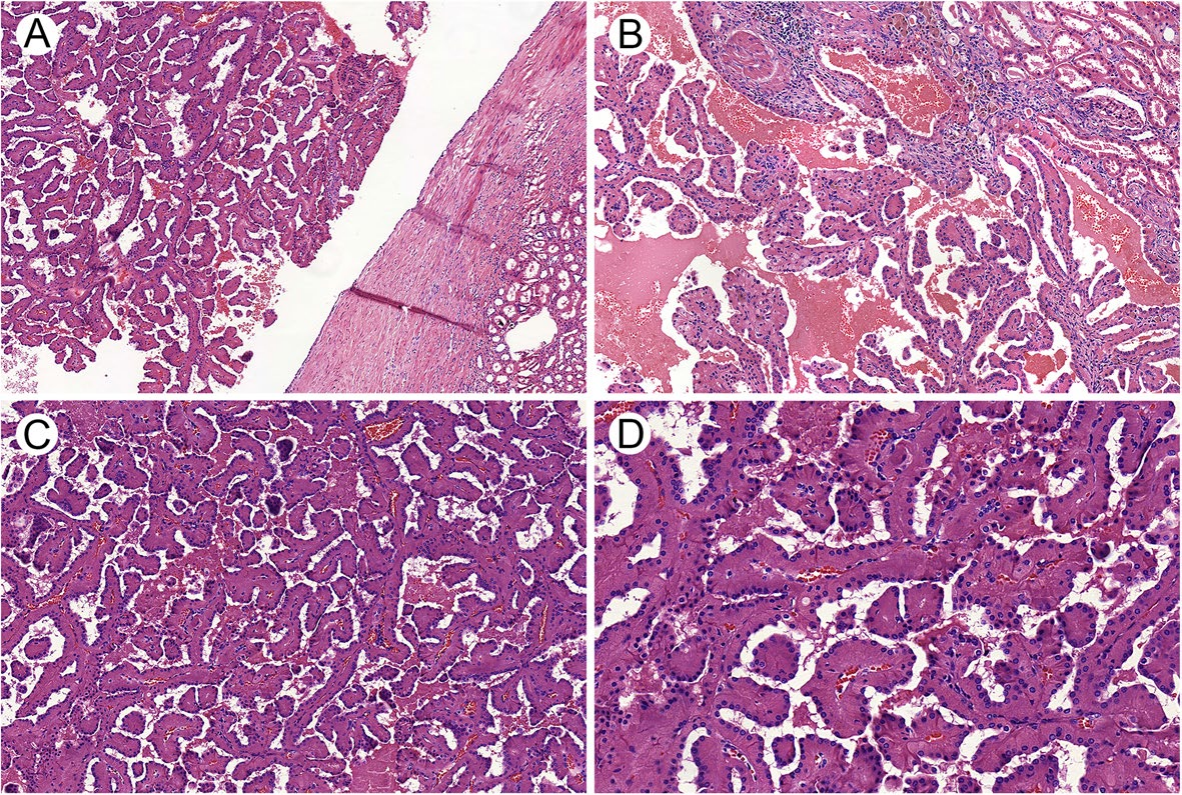
Histological characteristics of papillary renal neoplasm with reverse polarity. **A** Most of the tumors showed obvious intracystic papillary growth. **B** Transition zone between tumor tissue and renal tubular epithelia. **C** Tumors were primarily composed of papillary structures with complex branches and fibrovascular cores. **D** The tumor cells had abundant eosinophilic cytoplasm, with round nuclei far away from the basement membrane

(1) Gross morphology: the tumors were located in cortex of one of the renal poles (8 cases) or middle kidney (3 cases), all close to the renal capsule. The tumor diameter varied from 0.8 to 6 cm, with a median diameter of 2.8 cm. The cut surfaces of 8 tumors were solid and nodular, well-circumscribed, of which 5 were gray-yellow or gray-red and 3 cases had fine papillary appearance. Two tumors were cystic with 0.2–0.4 cm rough cyst wall, filled with yellow, thick curdy substance. One patient (Case 1) had bifocal lesions with cauliflower-like appearance protruding from the surface of kidney. One patient (Case 11) had bilateral renal tumors and underwent partial nephrectomy for the left renal tumor.

(2) Histological characteristics: Most of the PRNRP tumors had clear boundaries between the surrounding normal tissues. Intracystic papillary growth could be observed in 9 cases (Fig. 1A). Two tumors were directly adjacent to the surrounding renal tissues, with a transition between tumor tissue and renal tubular epithelia (Fig. 1B). All of the PRNRP tumors were predominantly formed by complex branching papillary structures with fibrovascular cores (Fig. 1C). Typically, the papillae were cover by a single layer of cuboidal tumor cells with medium amount of deep eosinophilic cytoplasm, unclear boundaries, and apically located small, round and regular nuclei without conspicuous nucleoli (Fig. 1D). According to the WHO/ISUP grading system, 10 cases were classified as grade 1 and 1 case as grade 2.

Additionally, 3 cases showed a small proportion of acini, tubular or cystic structures (Supplementary Fig. S1A). Focal solid area was observed in 1 case, with some tumor cells containing large transparent vacuoles in the cytoplasm (Fig. S1B). In 2 cases, large tumor cells with abundant, slightly eosinophilic or foam like cytoplasm were locally visible (Fig. S1C). In 5 cases, the stroma of tumors was scanty with clustered lymphocytes (Fig. S1D).

**Fig. 2:**
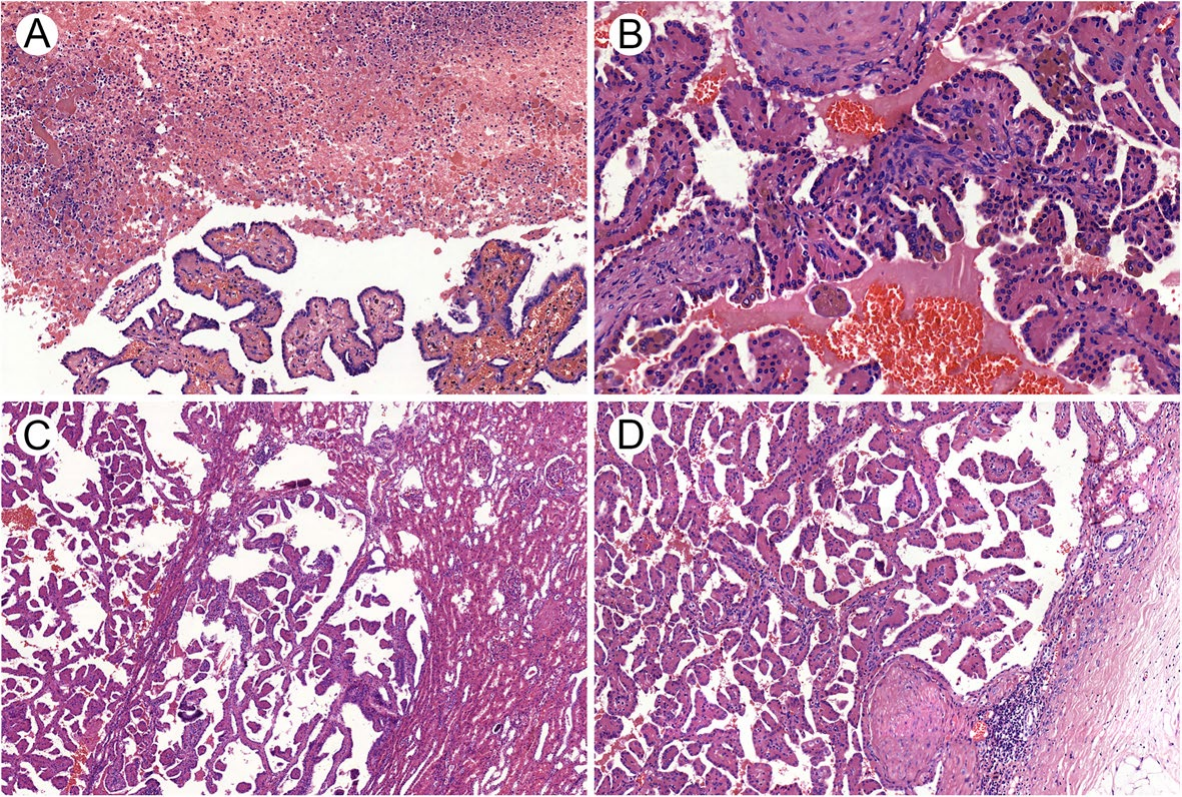
Some unusual morphological features of papillary renal neoplasm with reverse polarity. **A** Patchy necrosis in tumor tissue. **B** Hemosiderin deposits in the cytoplasm of tumor cells, especially in the edge of tumors. **C** Tumor components focally invaded into renal parenchyma. **D** One tumor exhibited perineural invasion

Notably, PRNRP demonstrated some unusual histological features (Fig. 2 and Supplementary Fig. S1), some of which has not yet been reported to our knowledge. Eight cases showed focal or extensive hemorrhage, and 5 had multifocal or patchy necrosis (Fig. 2A). Hemosiderin was deposited in the cytoplasm of a limited number of tumor cells in 6 cases, especially common at tumor margins (Fig. 2B). Moreover, 6 tumors exhibited small invasions into the cyst wall or renal parenchyma (Fig. 2C), and one tumor breached the renal capsule accompanied by nerve invasion (Fig. 2D). One tumor showed a pushing phenomenon on the adipose tissue in renal pelvis (Fig. S1E). Two cases exhibited local “hobnail” cells (Fig. S1F). Individual foam-like macrophages presented in the papilla core of 1 tumor (Fig. S1G). Besides, eosinophilic “multinucleated tumor cells” existed in all 11 tumors, with varying amounts and scattered distribution (Fig. S1H).

The clinicopathological data of 11 cases of PRNRP were presented in Table 1, and the results of statistical comparisons between PRNRP, type I PRCC and type II PRCC cases were listed in Table 2. The average diameter at onset of PRNRP was significantly smaller than that of type I PRCC (*P* = 0.004). Rate of hemosiderin deposits in cytoplasm was remarkably higher in PRNRP (6/11, in a small portion of tumor cells) and type I PRCC (7/16) than in type II PRCC (0/9, *P* = 0.014 and *P* = 0.027, respectively). Foam-like macrophages in PRNRP (1/11, individual cells) were significantly less than that in type I PRCC (11/16, *P* = 0.005). The proportion of patients with low WHO/ISUP nuclear grade was considerably higher than that of type I (11/16, *P* < 0.001) and type II PRCC patients (1/9, *P* < 0.001). In addition, the incidence of microvascular invasion in PRNRP (0/11) was markedly reduced compared with that of type II PRCC (4/9, *P* = 0.026).

**Table 1:** Clinicopathologic data of 11 patients with papillary renal neoplasm with reverse polarity

No	Gender	Age	No. of tumor	Diameter	Laterality	Necrosis	Hemorrhage	Infiltration	Microvascular invasion	Foamy macrophages	Intracellular hemosiderin	Clustered lymphocytes	Multinucleated tumor cells	WHO/ISUP nuclear grade	Stage	Follow-up Period (mo)	Status
1	F	67	2	3, 1.5	Left	no	focal	Breakthrough of renal capsule accompanied by nerve invasion	no	no	yes (few)	yes	yes	1	T1aN0M0	78	ADF
2	M	50	1	4	Left	patchy	multifocal	no	no	no	no	yes	yes	1	T1aN0M0	74	ADF
3	M	67	1	1.8	Right	no	obvious	focal	no	no	Yes (in the overlying epithelium of cyst)	no	yes	2	T1aN0M0	lost	—
4	F	62	1	1.7	Left	multifocal	focal	multifocal	no	no	yes (few)	yes	yes	1	T1aN0M0	103	ADF
5	M	63	1	3	Left	no	no	focal	no	no	yes (few)	no	yes	1	T1aN0M0	21	ADF
6	F	47	1	5	Right	extensive	extensive	no	no	no	no	no	no	1	T1bN0M0	63	ADF
7	M	26	1	6	Left	multifocal	focal	no	no	no	no	no	yes	1	T1bN0M0	19	ADF
8	M	63	1	0.8	Right	focal	no	focal	no	no	no	yes	yes	1	T1aN0M0	17	ADF
9	M	70	1	1.5	Left	no	yes	no	no	no	no	no	yes	1	T1aN0M0	6	ADF
10	F	64	1	1.2	Left	no	no	no	no	yes (few)	yes (few)	no	yes	1	T1aN0M0	17	ADF
11	M	60	1	2.3^a^	Bilateral	no	focal	focal	no	no	yes (few)	yes	yes	1	T1aN0M0	16	ADF

*Abbreviations:* *F* female, *M* male, *ADF* alive, disease-free

^a^Only left renal tumor was resected

**Table 2:** Comparison of clinicopathologic characteristics of papillary renal neoplasm with reverse polarity with type I and type II papillary renal cell carcinoma

	PRNRP (*n* = 11)	type I PRCC (*n* = 16)	type II PRCC(*n* = 9)	PRNRP vs type I PRCC	PRNRP vs type II PRCC	type I PRCC vs type II PRCC
*Gender (M: F)*	1.7:1	3:1	2:1	0.675	1.000	0.673
*Age (year)*	26–70 (mean 58)	30–74 (mean 57)	30–76 (mean 56)	0.957	0.943	0.996
*Diameter (cm)*	0.8 ~ 6 (2.8)	2.5 ~ 12 (5.7)	2.5 ~ 6 (4.8)	**0.004**	0.099	0.612
*Tumor cells*	Single-layered, eosinophilic	Single-layered, basophilic	Stratified, eosinophilic	—	—	—
*Inverted nuclei*	11/11	0/16	0/9	** < 0.001**	** < 0.001**	1.000
*Intracellular hemosiderin*	6/11 (few cells)	7/16	0/9	0.704	**0.014**	**0.027**
*Foamy macrophages in stroma*	1/11	11/16	4/9	**0.005**	0.127	0.397
*WHO/ISUP nuclear grade* (high vs. low)^a^	Grade 1: 10 Grade 2: 1	Grade 1: 2 Grade 2: 9 Grade 3: 5	Grade 2: 1 Grade 3: 6 Grade 4: 2	** < 0.001**	** < 0.001**	**0.011**
*Necrosis*	5/11	10/16	8/9	0.452	0.070	0.355
*Infiltrative growth*	6/11	7/16	6/9	0.704	0.670	0.411
*Microvascular invasion*	0/11	1/16	4/9	1.000	**0.026**	**0.040**
*Sarcomatoid differentiation*	0/11	1/16	0/9	1.000	1.000	1.000
*Stage* (early-stage vs. advanced-stage)^b^	Stage I: 11	Stage I: 10 Stage II: 3 Stage III: 3	Stage I: 8 Stage IV:1	0.248	0.450	1.000

Bold value indicates statistically significant

^a^WHO/ISUP Nuclear grades 1 and 2 were classified as low nuclear grade and Grades 3 and 4 were high nuclear grade

^b^TNM stage I and II were classified as early-stage and TNM stage III and IV were advanced-stage

### Immunohistochemical feature

**Fig. 3:**
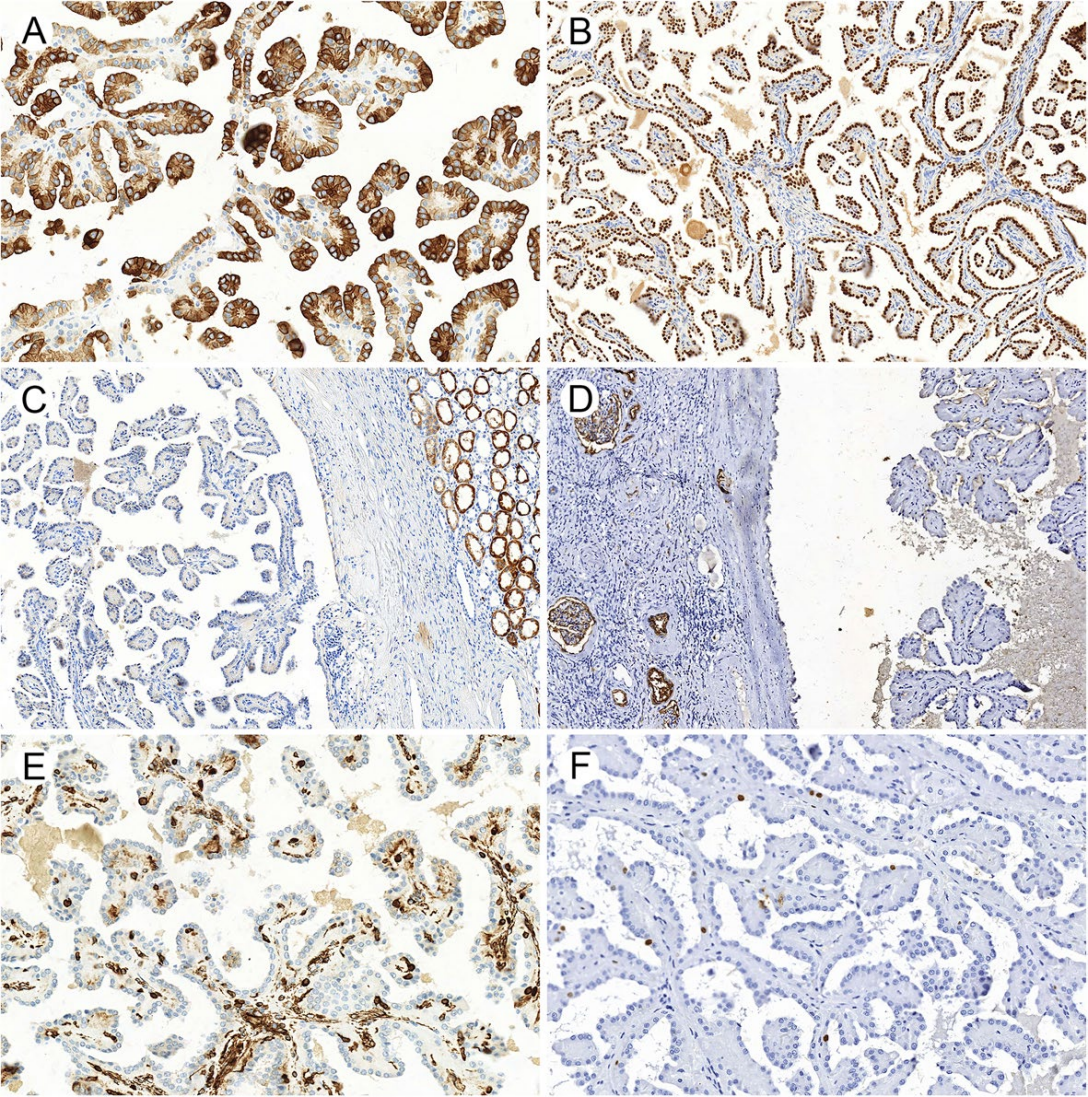
Immunohistochemical features of papillary renal neoplasm with reverse polarity. Tumors were consistently strongly positive for CK7 (**A**) and GATA3 (**B**), weakly positive or negative for P504S (**C**), and consistently negative for CD10 (**D**) and vimentin (**E**), with relatively low Ki-67 index (**F**)

All the 11 PRNRP cases showed strong and diffuse positive expression of CK7 (Fig. 3A), EMA, Pax8 and GATA3 (Fig. 3B, except 1 weakly positive). Most of the tumors were negative for P504S, with only 4/11 cases demonstrating weak (3 cases) or moderate (1 case) staining (Fig. 3C). CD10 was negative in 10/11 cases and weakly positive in only 1 tumor (Fig. 3D). All tumors showed negative staining for vimentin (Fig. 3E), CA9, CD117, RCC, TFE3, ALK, HMB45 and Melan A, with Ki67 index of 1–3% (Fig. 3F).

The immunohistochemical results of PRNRP, type I and type II PRCC cases were respectively shown in Supplementary Table S1, S2 and S3, and the corresponding statistical analysis were presented in Table 3. The positive rates of vimentin (*P* < 0.001 and *P* < 0.001), P504S (*P* = 0.003 and *P* = 0.005) and RCC (*P* < 0.001 and *P* = 0.002) in PRNRP were significantly lower than those in type I and type II PRCC, while GATA3 was expressed exclusively in PRNRP. Compared with type II PRCC, PRNRP exhibited a higher positive rate of CK7 (*P* = 0.008) and a reduced expression rate of CD10 (*P* < 0.001). There was no significant difference in the expression of EMA, CA9, CD117, TFE3 or ALK among the three tumor types.

**Table 3:** Comparison of immunophenotype of papillary renal neoplasm with reverse polarity with type I and type II papillary renal cell carcinoma

	PRNRP (*n* = 11)	type I PRCC (*n* = 16)	type II PRCC (*n* = 9)	PRNRP vs type I PRCC	PRNRP vs type II PRCC	type I PRCC vs type II PRCC
Vimentin	0/11	16/16	7/9	** < 0.001**	** < 0.001**	0.120
EMA	11/11	13/16	7/9	0.248	0.190	1.000
CK7	11/11	12/16	4/9	0.123	**0.008**	0.200
CD10	1/11	7/16	8/9	0.090	** < 0.001**	**0.041**
CA9	0/11	0/16	1/9	1.000	0.450	0.360
P504S	4/11	15/16	9/9	**0.003**	**0.005**	1.000
RCC	0/11	13/16	6/9	** < 0.001**	**0.002**	0.630
GATA3	11/11	0/16	0/9	** < 0.001**	** < 0.001**	1.000
PAX8	11/11	15/16	4/9	1.000	0.008	**0.012**
CD117	0/11	0/16	0/9	1.000	1.000	1.000
TFE3	0/11	1/16	0/9	1.000	1.000	1.000
ALK	0/11	0/16	0/9	1.000	1.000	1.000
Ki67 (%)	1 ~ 3	2 ~ 15	2 ~ 20			

Bold value indicates statistically significant

### KRAS and BRAF mutation detection

**Fig. 4:**
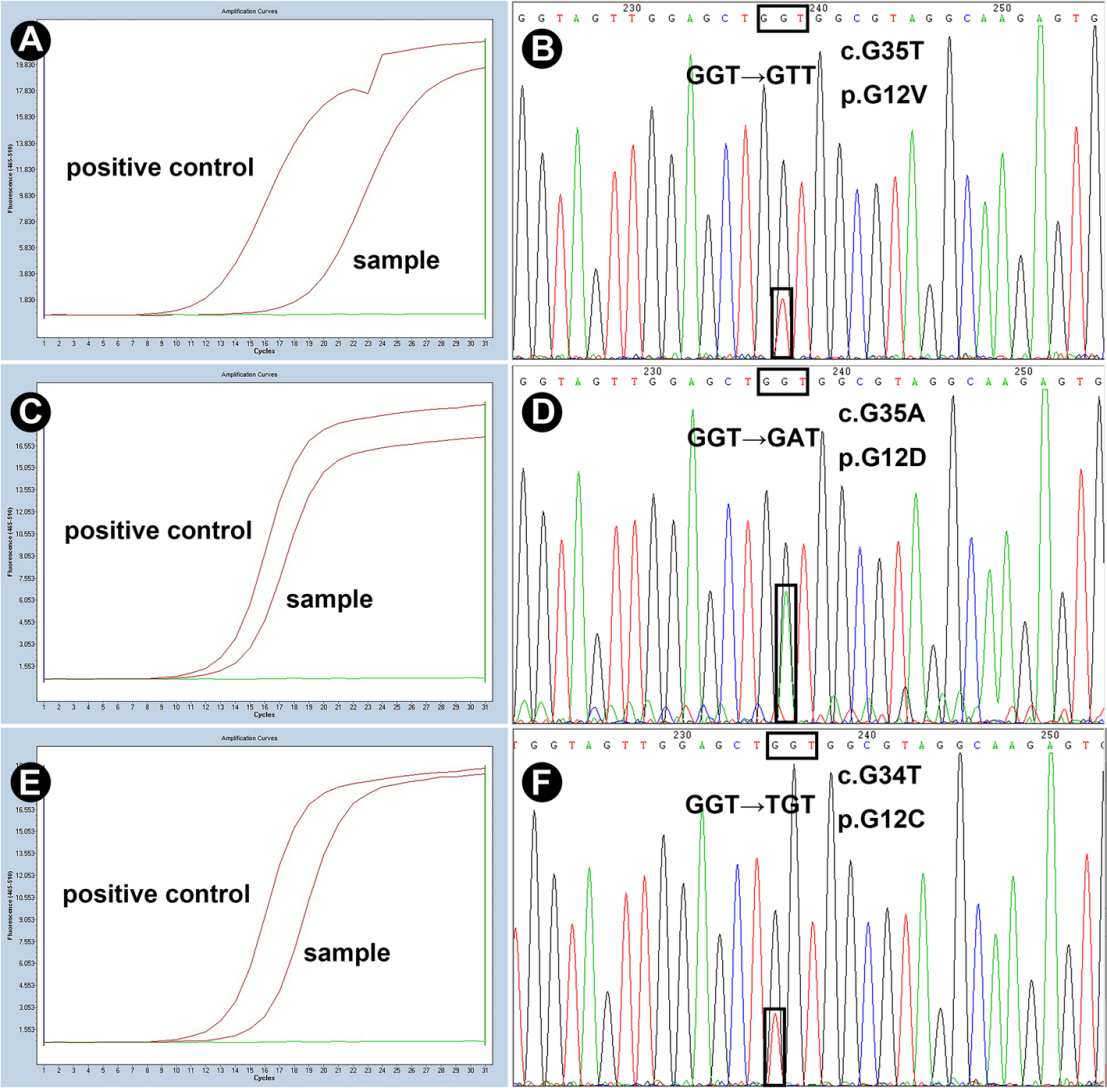
Detection of *KRAS* mutations in papillary renal neoplasm with reverse polarity. The positive amplification curves of real-time PCR analysis and the mutant peaks detected in Sanger sequencing are presented in left and the right panel, respectively. Representative results of G12V (**A** and **B**), G12D (**C** and **D**) and G12C (**E** and **F**) mutations are shown

(1) Real-time PCR analysis: *KRAS* mutations were detected in 10/10 cases of PRNRP, including 8 cases of G12C/G12R/G12V/G12A/G13C mutations (Fig. 4A and 4E) and 2 cases of G12S/G12D (Fig. 4C) mutations. No mutations in *NRAS*, *BRAF* or *PIK3CA* were confirmed. By contrast, 10 cases of PRCC (5 cases of type I and 5 cases of type II PRCC) were negative for any mutations in PCR detections.

(2) Sanger sequencing: Nine in 10 cases of PRNRP detected mutations located in *KRAS* exon 2, including 7 cases with G12V (Fig. 4B), 1 with G12D (Fig. 4D), and 1 with G12C mutation (Fig. 4F). One case failed sequencing due to insufficient quality of DNA samples. Detailed information was shown in Table 4. On the contrary, mutation in *KRAS* exon 2 was absent in all the 10 cases of PRCC (5 cases of type I and 5 cases of type II). No *BRAF* V600E mutation was detected in all cases.

**Table 4:** Molecular genetic features of papillary renal neoplasm with reverse polarity

No	Gender	Gene mutations		Chromosomal abnormalities		
		*KRAS* mutation	*BRAF* V600E	Chromosome 7 trisomy	Chromosome 17 trisomy	Chromosome Y deletion
1	F	G12V	-	-	-	female
2	M	sequencing failure	sequencing failure	NA	NA	NA
3	M	NA	NA	NA	NA	NA
4	F	G12V	-	NA	NA	NA
5	M	G12V	-	-	-	-
6	F	G12V	-	NA	NA	NA
7	M	G12D	-	-	-	-
8	M	G12V	-	+	-	-
9	M	G12V	-	-	-	-
10	F	G12V	-	-	-	female
11	M	G12C	-	-	-	-

*Abbreviations*: *NA* not available

### FISH results

Chromosome 7 trisomy (1/7 cases), chromosome 17 trisomy (0/7 cases), and chromosome Y deletion (0/5 male cases) were almost absent in PRNRP cases. Further details are in Table 4 and typical images of FISH are shown in Supplementary Fig. S2.

### Follow-up data

Ten of 11 patients with PRNRP were followed up for 6–103 months (median 20 months, average 41 months). One (Case 11) was complicated with small cell lung cancer and underwent radical surgery, and another patient (Case 1) was diagnosed with cervical cancer 3 years after surgery and received conservative treatment. All patients were alive without recurrence or metastasis of renal tumor as of their most recent follow-up.

## Discussion

The morphological feature of inverted nuclear polarity had been reported in previous literatures, without attracting special attention for a long time. According to the study of Lefèvre et al. [12] in 2005, at least 1 out of 10 cases of so-called oncocytic PRCC exhibited inverted nuclei in published histological images. In 2008, Kunju et al. [13] described 7 cases of PRCC with oncocytic cells and nonoverlapping low grade nuclei, of which some cases also showed inverted nuclei. One year later, Park et al. [14] for the first time summarized 7 cases of oncocytic PRCC with inverted nuclear pattern. In 2017, Saleeb et al. [15] proposed a subtype of PRCC (Type 4) with low-grade non overlapping nuclei arranged in a special linear pattern on the opposite side of the basement membrane. In 2019, Al-Obaidy et al. [7] summarized 18 cases of papillary renal tumors with similar histological features and proposed the name “papillary renal neoplasm with reverse polarity” for this tumor entity. The diagnostic criteria of the tumor were described as follows: papillary or tubular structure, lined by a single layer of eosinophilic cells with fine granular cytoplasm, round and apically located nuclei and unclear nucleoli, without intracellular hemosiderin, necrosis or mitotic figures [7]. Several subsequent studies also reported that no necrosis was found in PRNRP [8, 9, 16, 17].

Different from the aforementioned criteria, in this study, some of the PRNRP cases showed focal to patchy necrosis and infiltrative tumor margins. Although hemosiderin in cytoplasm of the tumor cells were not as common as those in type I PRCC, a small number of intracellular hemosiderin deposits were still observed. In addition, scattered eosinophilic “multinuclear tumor cells” existed in all PRNRP cases. Perhaps they are sectional profiles of the papillae, but this morphology is conspicuous and has not yet been reported, and might act as a cue in the diagnosis of PRNRP.

PRNRP was considered to have a favorable prognosis, and no tumor-related death was reported [7–9, 16, 17]. In this study, 10 patients were followed up for 6 to 104 months, and all survived without metastasis or recurrence at the end of the follow-up. Notwithstanding the varying degrees of necrosis or irregular invasive margins observed in our cases, we still tent to consider PRNRP as a tumor entity with low malignant potential. Due to the limited number of reported cases and the lack of long-term follow up data, the biological behavior of this tumor needs further in-depth study.

The immunophenotype of PRNRP is characterized by positive staining of CK7, EMA, and GATA3, variable but low expression of P504S, and negative staining of vimentin and CD10. Based on the literature and our results, the immunohistochemical features of PRNRP overlap with those of types I and II PRCC, but still present some unique characteristics. Vimentin, CD10 and RCC, usually positive in PRCC [2, 18, 19], were almost absent in PRNRP. P504S, as a commonly used positive immunomarker of PRCC [20], was seldom or weakly expressed in PRNRP. The expression of GATA3 in PRNRP was constant (100%), and most of the cases exhibited diffusely strong staining, distinctly different from the complete negative expression in type I and type II PRCC.

GATA3, as a member of GATA transcription factor family, regulated by Pax-2/Pax-8, mediates the development of pronephron and mesonephron, and plays a key role in guidance of the nephric duct [21]. GATA3 haploinsufficiency participates in the pathogenesis of the hypoparathyroidism, sensorineural deafness and renal dysplasia (HDR) syndrome [22]. In view of the fact that the regulatory factors of some embryonic development stages are also closely related to tumorigenesis, it is speculated that GATA3 might participate in the tumor initiation or progression of PRNRP. However, a clear mechanism of its function has not been firmly established.

*KRAS* mutation is an important molecular event recently discovered in PRNRP. As one of the most frequently activated oncogenes, *KRAS* mutations were reported to exist in 17%—25% of human tumors [23], most of which were detected in codons 12 and 13, including different point mutations, such as G12S, G12D, G12C, G12V and G13D. Since 2019, several studies have reported *KRAS* mutation in PRNRP, and the results are highly consistent [8, 16, 24]. Due to the rarity of *KRAS* mutation in RCC and its high incidence in PRNRP, *KRAS* mutation has become one of the most representative molecular markers that distinguish PRNRP from other PRCC subtypes, and it is likely to be a key driving factor in the tumorigenesis of this type of tumor. In addition, targeted second-generation sequencing detected sporadic *BRAF* V600E mutation in rare PRNRP cases [24], but no PRNRP case with concomitant *BRAF* V600E mutation and *KRAS* mutation was found either in this study or in prior reports, which is consistent with the understanding that *BRAF* mutations are mutually exclusive with KRAS mutations [25].

As is well known, chromosome 7 trisomy, chromosome 17 trisomy and chromosome Y deletion are characteristic genetic features of PRCC, yet type I and type II PRCC represent different genetic features. According to a comprehensive study on molecular characteristics of renal cell carcinoma based on The Cancer Genome Atlas, most type I PRCC showed gains of chromosome 7 and 17, while the incidence of such chromosomal abnormalities in type II PRCC was about 20–30% [26]. In our study, chromosome 7 trisomy, chromosome 17 trisomy, and chromosome Y deletion were barely detected in PRNRP patients, suggesting that PRNRP may be a unique subtype of renal cell neoplasm, independent from type I or type II PRCC.

In conclusion, our study presents some novel morphological manifestations of PRNRP. The good agreement with the reported immunohistochemical characteristics and the high incidence of *KRAS* mutations in exon 2 supported our identification and inclusion of PRNRP cases. Due to the papillary growth pattern, favorable survival outcomes, and the low presence of characteristic chromosomal abnormalities for PRCC, we suggest that this tumor entity be considered as a new independent renal cell neoplasm with low malignant potential. Its long-term biological behavior, as well as the role of *KRAS* mutations and the high expression of GATA3 in tumorogenesis and progression, are expected to be further understood.

## Supplementary Information

**Additional file 1: Fig. S1.** Other histologic appearance of papillary renal neoplasm with reverse polarity. (A) Tumors were well circumscribed, with different proportions of tubular or cystic architectures. (B) One tumor had focal solid area with large transparent vacuoles in the cytoplasm of some tumor cells. (C) Most of the tumor cells were medium-sized and deeply eosinophilic, interspersed with a small number of large cells with abundant slightly eosinophilic or foamy cytoplasm. (D) Cluster-like lymphocytic aggregations in stroma were detectable. (E) One tumor appeared to exhibit a pushing phenomenon on the adipose tissue in renal pelvis. (F) Some tumor cells represented a “hobnail” morphology. (G) Foam-like macrophages (black arrow) in the papilla core were hardly observed.(H) Scattered “multinucleated tumor cells” (black arrows) in tumor stroma.

**Additional file 2: Fig.S2.** Representative FISH results. Except for one patient (Case 8) with chromosome 7 trisomy (A), other cases did not show gains of chromosome 7 (B) or 17(C), or the deletion of Y chromosome (D).

**Additional file 3:** Supplementary Tables.

**Additional file 4:** Supplementary Materials and Methods.
