# Supplementary figures and images for: Papillary renal neoplasm with reverse polarity may be a novel renal cell tumor entity with low malignant potential
Source: Diagn Pathol. 2022 Aug 25;17:66. doi: 10.1186/s13000-022-01235-2 (PMC9404576; doi:10.1186/s13000-022-01235-2)

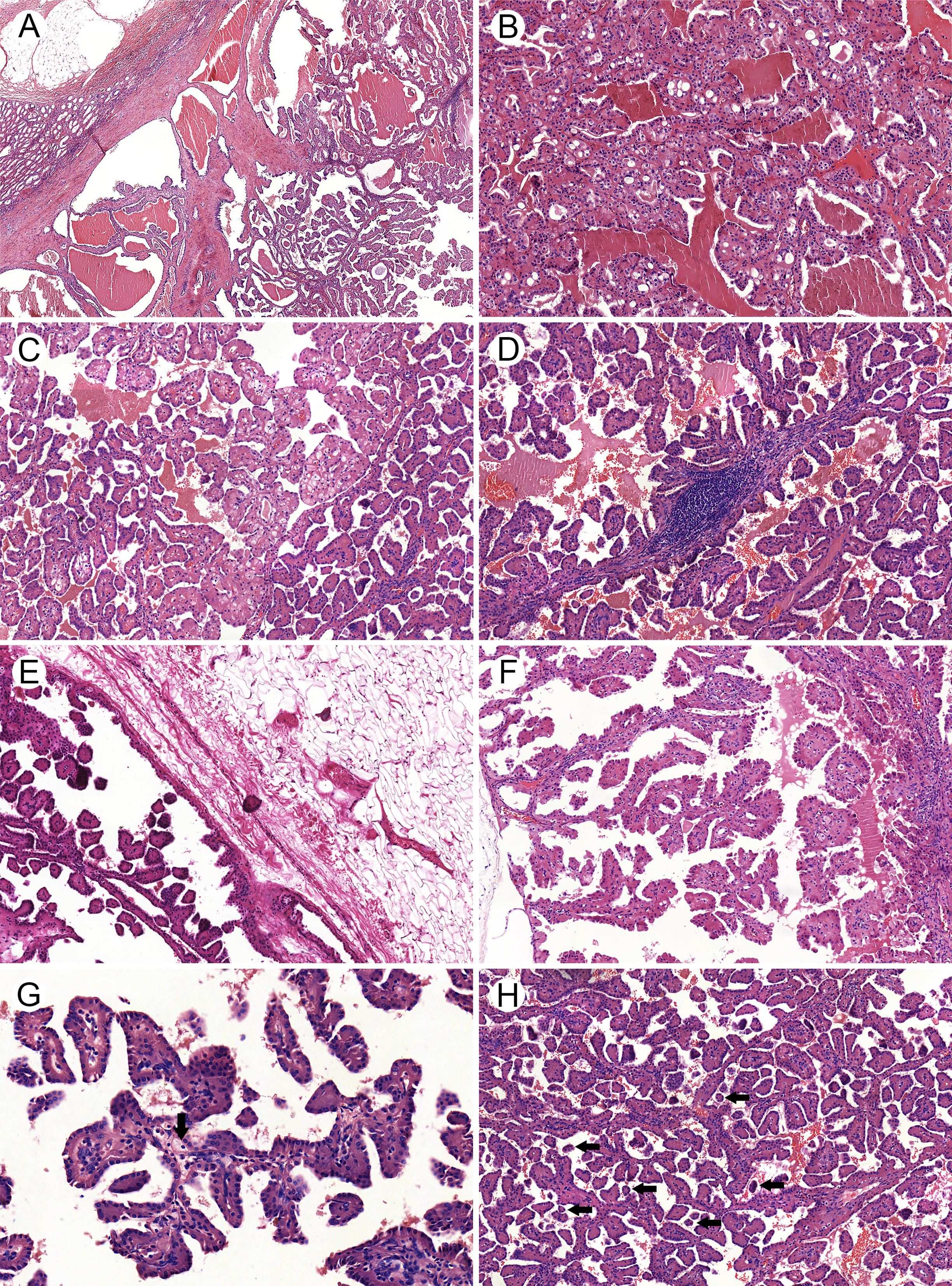

Supplement: Supplementary file 1 — Additional file 1: Fig. S1. Other histologic appearance of papillary renal neoplasm with reverse polarity. (A) Tumors were well circumscribed, with different proportions of tubular or cystic architectures. (B) One tumor had focal solid area with large transparent vacuoles in the cytoplasm of some tumor cells. (C) Most of the tumor cells were medium-sized and deeply eosinophilic, interspersed with a small number of large cells with abundant slightly eosinophilic or foamy cytoplasm. (D) Cluster-like lymphocytic aggregations in stroma were detectable. (E) One tumor appeared to exhibit a pushing phenomenon on the adipose tissue in renal pelvis. (F) Some tumor cells represented a “hobnail” morphology. (G) Foam-like macrophages (black arrow) in the papilla core were hardly observed.(H) Scattered “multinucleated tumor cells” (black arrows) in tumor stroma. [file 13000_2022_1235_MOESM1_ESM.jpg]

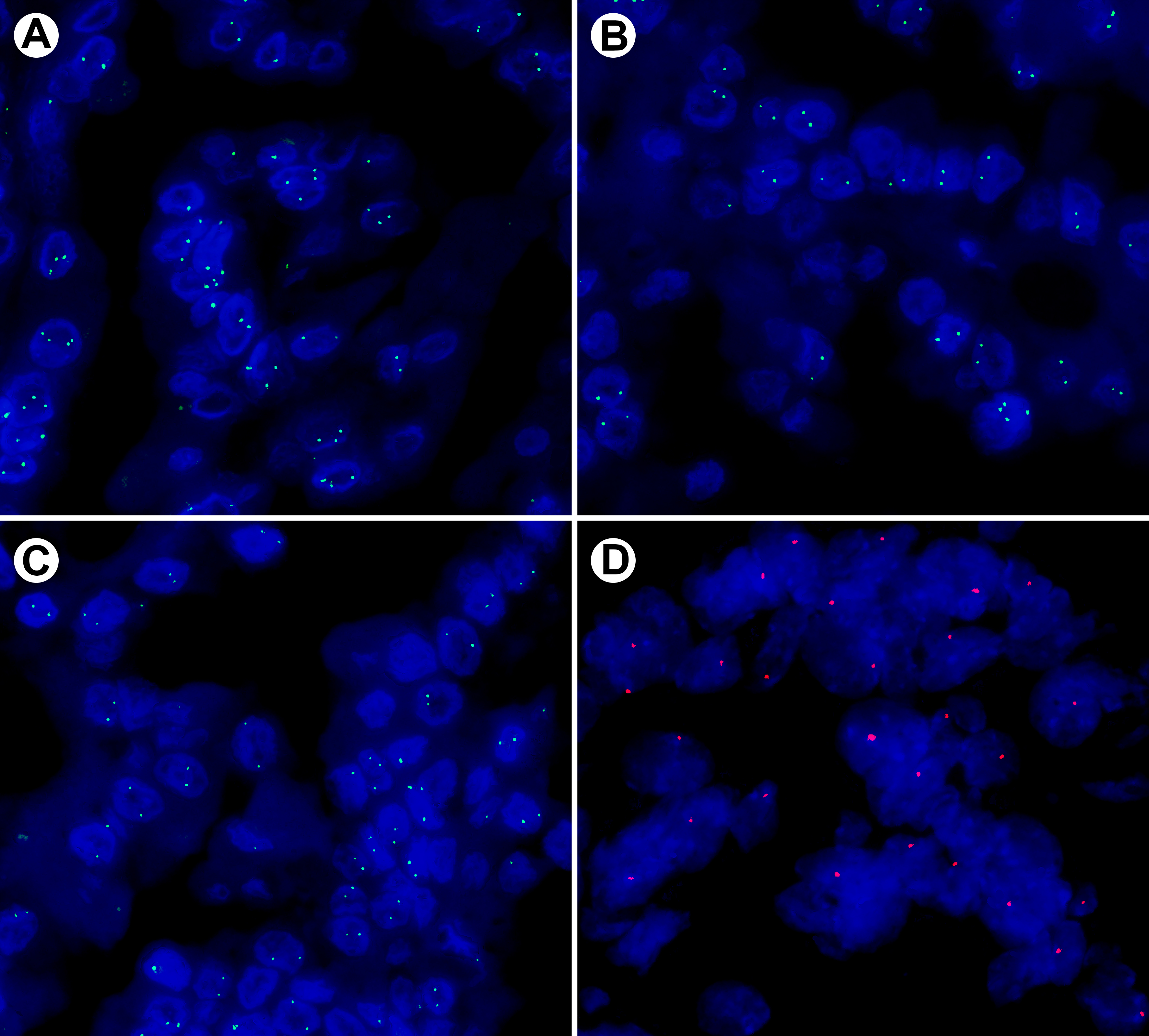

Supplement: Supplementary file 2 — Additional file 2: Fig.S2. Representative FISH results. Except for one patient (Case 8) with chromosome 7 trisomy (A), other cases did not show gains of chromosome 7 (B) or 17(C), or the deletion of Y chromosome (D). [file 13000_2022_1235_MOESM2_ESM.jpg]
