# Supplementary material for: Papillary renal neoplasm with reverse polarity may be a novel renal cell tumor entity with low malignant potential
Source: Diagn Pathol. 2022 Aug 25;17:66. doi: 10.1186/s13000-022-01235-2 (PMC9404576; doi:10.1186/s13000-022-01235-2)
Supplement: Supplementary file 3 — Additional file 3: Supplementary Tables. [file 13000_2022_1235_MOESM3_ESM.docx]

**Supplementary Table S1** Immunohistochemical features of 11 cases of papillary renal neoplasm with reverse polarity

| No. | **vimentin** | **EMA** | **CK7** | **CD10** | **CA9** | **P504S** | **RCC** | **PAX8** | **CD117** | **CK20** | **GATA3** | **TFE3** | **ALK** |
| --- | --- | --- | --- | --- | --- | --- | --- | --- | --- | --- | --- | --- | --- |
| 1 | - | +++ | +++ | - | - | - | - | +++ | - | - | +++ | - | - |
| 2 | - | +++ | ++ | - | - | - | - | ++ | - | - | +++ | - | - |
| 3 | - | +++ | +++ | - | - | - | - | ++ | - | - | +++ | - | - |
| 4 | - | +++ | +++ | - | - | ++ | - | ++ | - | - | + | - | - |
| 5 | - | +++ | +++ | + | - | - | - | +++ | - | - | +++ | - | - |
| 6 | - | +++ | +++ | - | - | + | - | ++ | - | - | ++ | - | - |
| 7 | - | +++ | +++ | - | - | + | - | +++ | - | - | +++ | - | - |
| 8 | - | +++ | +++ | - | - | + | - | ++ | - | - | +++ | - | - |
| 9 | - | +++ | ++ | - | - | - | - | +++ | - | - | ++ | - | - |
| 10 | - | +++ | +++ | - | - | - | - | +++ | - | - | +++ | - | - |
| 11 | - | +++ | +++ | - | - | - | - | +++ | - | - | +++ | - | - |

**Supplementary Table S2** Immunohistochemical features of 16 cases of Type I PRCC.

| **No.** | **vimentin** | **EMA** | **CK7** | **CD10** | **CA9** | **P504S** | **RCC** | **PAX8** | **CD117** | **GATA3** | **TFE3** |
| --- | --- | --- | --- | --- | --- | --- | --- | --- | --- | --- | --- |
| 1 | +++ | +++ | +++ | - | - | - | - | +++ | - | - | - |
| 2 | +++ | - | - | ++ | - | +++ | - | ++ | - | - | - |
| 3 | +++ | ++ | +++ | - | - | ++ | +++ | ++ | - | - | - |
| 4 | +++ | - | - | ++ | - | +++ | +++ | +++ | - | - | ++  (focal) |
| 5 | ++ | +++ | +++ | ++  (focal) | - | +++ | - | +++ | - | - | - |
| 6 | +++ | +++ | +++ | - | - | +++ | +++ | ++  (focal) | - | - | - |
| 7 | +++ | +++ | +++ | + | - | +++ | +++ | +++ | - | - | - |
| 8 | +++ | +++ | - | - | - | +++ | ++ | ++ | - | - | - |
| 9 | +++ | +++ | +++ | ++  (focal) | - | +++ | ++ | +++ | - | - | - |
| 10 | +++ | +++ | +++ | - | - | +++ | +++ | ++ | - | - | - |
| 11 | +++ | +++ | +++ | - | - | +++ | + | ++ | - | - | - |
| 12 | ++ | +++ | ++ | - | - | ++ | +++ | ++ | - | - | - |
| 13 | +++ | +++ | +++  (focal) | - | - | +++ | +++ | +++ | - | - | - |
| 14 | + | +++ | +++ | - | - | +++ | ++ | - | - | - | - |
| 15 | +++ | - | - | ++ | - | +++ | +++ | ++ | - | - | - |
| 16 | +++ | +++ | +++ | ++  (multifocal) | - | +++ | ++ | ++ | - | - | - |

**Supplementary Table S3** Immunohistochemical features of 9 cases of Type II PRCC.

| **No.** | **vimentin** | **EMA** | **CK7** | **CD10** | **CA9** | **P504S** | **RCC** | **PAX8** | **CD117** | **GATA3** | **TFE3** |
| --- | --- | --- | --- | --- | --- | --- | --- | --- | --- | --- | --- |
| 1 | - | +++ | - | - | - | +++ | - | - | - | - | - |
| 2 | - | ++ | - | + | - | +++ | +++ | - | - | - | - |
| 3 | +++ | +++ | - | ++ | - | +++ | + | +++ | - | - | - |
| 4 | +++ | +++ | ++ | ++ | +  (focal) | +++ | ++ | + | - | - | - |
| 5 | +++ | - | - | +++ | - | +++ | +++ | ++ | - | - | - |
| 6 | +++ | ++ | - | +++ | - | +++ | - | - | - | - | - |
| 7 | +++ | +++ | +  (scattered) | +++ | - | +++ | ++ | - | - | - | - |
| 8 | +++ | +++ | ++  (scattered) | +++ | - | +++ | - | - | - | - | - |
| 9 | +++ | - | ++  (scattered) | +++ | - | ++ | ++ | ++ | - | - | - |
