# Supplementary material for: Papillary renal neoplasm with reverse polarity may be a novel renal cell tumor entity with low malignant potential
Source: Diagn Pathol. 2022 Aug 25;17:66. doi: 10.1186/s13000-022-01235-2 (PMC9404576; doi:10.1186/s13000-022-01235-2)
Supplement: Supplementary file 4 — Additional file 4: Supplementary Materials and Methods. [file 13000_2022_1235_MOESM4_ESM.docx]

**Supplementary Materials and Methods**

***Real-time PCR***

Five to eight sections, each 5 μm thick, were cut from each paraffin-embedded tumor sample of 10 PRNRP cases (except Case 3), 5 type I and 5 type II PRCC cases. Genomic DNA was isolated from these tissue sections by using a FFPE genomic DNA extraction kit (Tiangen Biotech Co. Ltd, Beijing, China). For primary detection of hotspot mutations, DNA concentration was measured on a Q3000 Micro-Ultraviolet Spectrophotometer (Quawell, Sunnyvale, CA, USA), and adjusted to 1.5-3 ng/μl. Thereafter, real-time qPCR was carried out by using a human *KRAS/NRAS/PIK3CA/BRAF* gene mutation joint detection kit (AmoyDx, Xiamen, Fujian, China) on a Cobas z480 platform (Roche, Rotkreuz, Switzerland). The preparation of reaction system and the interpretation of mutational signals were based on the manufacturer's instructions.

***Sanger Sequencing***

To further confirm the mutations detected by PCR, Sanger sequencing was performed. Full-length *KRAS* exon 2 and *BRAF* exon 15 were amplified by PCR using the extracted genomic DNA. The primers were designed using primer5 software. The following primers were used: *KRAS* forward primer sequence: 5 '-gcgtcgatggagggatttgt-3'; reverse primer sequence: 5’-TGGTCCTGCACCAGTAATATGC-3’. *BRAF* forward primer sequence: 5’-GGAAAGCATCTCACCTCATCCT-3’; reverse primer sequence: 5’-AGCCTCAATTCTTACCATCCACAAA-3’. The PCR cycling program was: step 1, 94°C 4 min; step 2, 94°C 20 s; step 3, 60°C 20 s; step 4, 72°C 40 s; repeat steps 2, 3 and 4 39 times; step 5, 72°C 10 min. PCR products were identified by agarose gel electrophoresis, purified and subsequently sequenced by Shenggong Bioengineering Technology Limited (Shanghai, China) using an ABI 3730XL DNA analyzer (Applied Biosystems, Foster City, CA, USA).

***Fish analysis***

Chromosome 7 probe (CEP7/D7Z1, Spectrum Green), chromosome 17 probe (CEP17/D17Z1, Spectrum Green) and chromosome Y probe (CEPY/DYZ3, Spectrum Orange) were purchased from Abbott Laboratories Trading Co,.Ltd (Shanghai, China). The analysis was conducted according to the manufacturer’s instructions. At least 100 non-overlapping nuclei were counted in each sample under the high magnification field, and normal renal tissue served as a control. The threshold value was defined as the mean value of signal number in non-tumor tissues (M) + three times the standard deviation (3 × SD). Samples with more than 15% of the tumor cells showing three or more green signals were interpreted as chromosome 7 or 17 trisomy.
